# Global trajectory and spatiotemporal epidemiological landscape of multidrug-resistant tuberculosis of spanning 46 years (1990–2035): implications for achieving global end TB goals

**DOI:** 10.3389/fpubh.2025.1660984

**Published:** 2025-09-30

**Authors:** Yunbin Yang, Aoran Yang, Renzhong Li, Wei Su, Jiawen Jiang, Liangli Liu, Yunzhou Ruan, Lin Xu

**Affiliations:** ^1^Division of Tuberculosis Control and Prevention Yunnan Center for Disease Control and Prevention, Kunming, China; ^2^Chongqing Three Gorges Medical College, Chongqing, China; ^3^Chinese Center for Disease Control and Prevention, Beijing, China

**Keywords:** multidrug-resistant tuberculosis, global, incidence, death, disability-adjusted life years

## Abstract

**Background:**

Although MDR-TB is recognized as a significant threat, systematic descriptions of its long-term (>30 years) global spatiotemporal evolution patterns are still limited.

**Objectives:**

This study conducted a 46-year spatiotemporal analysis of global MDR-TB (1990–2035) to provide key evidence for evaluating and refining the WHO End TB Strategy.

**Methods:**

We used Global Burden of Disease data to identify identified temporal inflection points in ASIR, ASDR, and DALYs using Joinpoint regression. Spatial clustering was quantified using Moran’s I and Getis-Ord hotspot analysis. A Bayesian age-period-cohort model projected MDR-TB incidence from 2022 to 2035.

**Results:**

The male-to-female ratio was approximately 1.5:1. Incidence was highest at 30–60 years, deaths at 60+, DALYs peak at 45–60; children under 14 years of age significantly affected. ASIR rose from 0.97/100 k (1990) to 6.39/100 k (2000), then declined (APC: −3.15%) post-2005 to 5.62/100 k (2021); males exhibited a sharper increase (+2.39%) and slower decline (−0.71%). ASDR peaked at 2.12/100 k (2002; males 27% higher). DALYs peaked at 89.05/100 k (2003). Sub-Saharan Africa is hyperendemic (Moran’s I = 12.38, *p* < 0.001; Somalia: 57.25/100 k), with high-high clusters in Africa/Kyrgyzstan. Projections: Global ASIR declines modestly (−1.62% by 2035), but 480,000 cases expected due to population growth; female incidence drops 7.27% (2025+), male trends stable.

**Conclusion:**

MDR-TB has proven more challenging than anticipated, with persistent hotspots in sub-Saharan Africa and a disproportionate impact on males, the older adults, and children. Despite a marginal decline in ASIR to 5.46 per 100,000, the absolute number of cases is projected to rise to 480,000 by 2035 due to sustained population growth and aging. This will seriously hinder the WHO End TB Strategy. Addressing MDR-TB should prioritize key populations and regions, targeted resources, tailored interventions, sustained investment in diagnostics and treatment, and stronger government support for patient care.

## Background

Multidrug-resistant tuberculosis (MDR-TB), defined by resistance to rifampicin and isoniazid—the two most effective first-line anti-TB drugs—continues to pose a critical global public health challenge. Effective management of MDR-TB requires prolonged treatment with costly second-line regimens that involve complex administration protocols and rigorous monitoring (1, 2). Despite overall global declines in tuberculosis incidence and death, the prevalence of MDR-TB has been increasing (3). The 2024 Global TB Report by World Health Organization (WHO) estimated 10.8 million new TB cases (representing an incidence of 134 per 100,000 population) and 1.25 million TB-related deaths in 2023. These figures surpass deaths from Coronavirus Disease 2019 (COVID-19) and reestablish tuberculosis as the foremost infectious disease killer (4). Achieving the targets of the WHO End TB goal—an 80% reduction in incidence, a 90% decline in death, and the elimination of catastrophic costs for affected families by 2030—remains a major challenge (5).

Among newly diagnosed TB cases, 400,000 were identified as MDR-TB, with resistance observed in 3.2% of new cases and 16% of re-treatment cases (4). Further, significant geographic disparities in MDR-TB epidemiology persist, especially in high-TB-burden nations such as India, the Philippines, China, Russia, and South Africa, where an increasing incidence of MDR-TB is projected despite improvements in acquired drug resistance management (6). Modeling studies anticipate substantial increases in MDR-TB cases in these countries by 2040, thereby underscoring the urgent need for enhanced containment strategies to interrupt transmission (7).

Although MDR-TB is recognized as a significant threat, systematic descriptions of its long-term (>30 years) global spatiotemporal evolution patterns is still limited. Existing research focuses on specific regions, short time span (such as after 2000) or single index (such as incidence rate), and lacks panoramic and long-term scale analysis integrating incidence rate, death, disability-adjusted life years (DALYs) and their age gender geographical heterogeneity. Song (8) focused on 30-year trends, but their analysis mainly focused on the comparison of overall trends and drug sensitivity TB. Lv (9) provided valuable global burden assessments, but their analysis time frame up to 2019 did not capture potential key turning points before and after the COVID-19 pandemic, and lacked depth in spatial heterogeneity and future predictions. Alene et al. (10) conducted research on northwestern Ethiopia, using spatial autocorrelation (Moran’s I) and local spatial association index (LISA) to identify MDR-TB hotspots, but only covered a single country or region and did not expand globally. Sharma et al.’s (7) prediction was limited to four high burden countries. Although Guo et al. (11) used GBD 2021 data, they only used ARIMA models to predict until 2030, without integrating population structure and cohort effects.

Therefore, building on previous research in this field in the early stage, this study has filled some key analysis gaps. It aims to conduct a comprehensive spatiotemporal epidemiological study of global MDR-TB for the first time over a period of 46 years (from 1990 and predicting until 2035) using the latest released GBD 2021 data. We use Joinpoint regression to accurately identify the turning point of the historical trend over the past 30 years. By analyzing the distribution characteristics of key populations through subgroup analysis, such as specific age groups and genders, describe the country clustering of MDR-TB and its spatio-temporal changes in combination with spatial autocorrelation analysis and identify the continuous high burden hotspots. The Bayesian Age Period Cohort (BAPC) model was applied to integrate historical epidemiological data and the United Nations population projections, accounting for future demographic and age-structural changes across different countries and regions. This model was selected not only for its ability to capture temporal trends in the data, but more importantly, for its incorporation of population characteristics (such as age structure, birth cohort effects, and demographic shifts) across different countries and regions, thereby providing a more nuanced and accurate reflection of future disease burden than traditional time-series forecasting methods—enabling more refined predictions of the global incidence rate and number of MDR-TB cases between 2022 and 2035. The projections provide critical evidence for assessing the feasibility and challenges of achieving the WHO End TB goals (12, 13).

## Methods

### Data source

This study leveraged multiple publicly accessible databases. Historical data on global MDR-TB incidence and death from 1990 to 2021 were extracted from the Global Burden of Disease (GBD) database,^1^ a comprehensive epidemiological repository managed by the Institute for Health Metrics and Evaluation (IHME) at the University of Washington. The GBD database systematically integrates globally representative estimates of diseases, injuries, and risk factors, including incident cases, deaths, and stratified metrics by age, gender, and geography across more than 200 countries and territories (14–16). To ensure consistency in geographic attribution, all region-specific data were aligned using ISO 3166-1 alpha-3 country codes. Demographic data were obtained from the United Nations World Population Prospects 2024 (WPP 2024),^2^ which provides historical population estimates (1950 to 2021) and projections (until 2,100) for age-structure standardization. For the standardization of age structure in incidence projections (2022 to 2035), the World Health Organization’s 2000 to 2025 Standard Population^3^ was applied, defining 5-year age intervals (0–4, 5–9, …, 100+) with corresponding weighting coefficients (17, 18). Geospatial boundaries were sourced from the Natural Earth dataset,^4^ a validated global administrative division database ensuring geopolitical neutrality (19). To harmonize geographic units across all datasets, we used the Natural Earth vector boundaries as the spatial reference. Regions with missing or low-quality data were explicitly flagged and excluded from high-resolution spatial analyses. Primary outcomes included the age-standardized incidence rate (ASIR), age-standardized death rate (ASDR), and DALYs associated with MDR-TB (excluding extensively drug-resistant tuberculosis [XDR-TB]), all reported with 95% confidence intervals (CI).

### Analytical methods for MDR-TB Ttrends, projections

#### Joinpoint regression

To examine temporal trends in MDR-TB ASIR, ASDR, and DALYs from 1990 to 2021, Joinpoint regression analysis was performed using the Joinpoint Regression Program (version 5.3.0, National Cancer Institute). This method identifies significant inflection points in epidemiological trajectories by iteratively fitting segmented log-linear regression curves. The optimal number of joinpoints (up to a maximum of five, allowing for six trend segments) was determined using permutation tests (4,500 iterations) to minimize the Bayesian Information Criterion (BIC) (20, 21). The annual percent change (APC) for each segment was calculated from the slope of the log-transformed rates using the formula APC = 100% × (e^*β* − 1), where β denotes the regression coefficient (22). Statistical significance was determined at *p* < 0.05. Trend directionality was inferred based on the 95% CI; a positive trend was defined by a lower bound>0, whereas a negative trend was indicated by an upper bound<0. Overlapping intervals were interpreted as stable trends.

#### Spatial auto-correlation analysis

Spatial autocorrelation analysis was conducted to identify geographic clustering of MDR-TB incidence. Global spatial dependency was assessed using Moran’s I statistic in ArcGIS 10.2 (Esri), with spatial weights defined by Queen contiguity (shared borders/vertices) (23, 24). Significance was determined through Monte Carlo randomization simulations (*p* < 0.05). Further, local clustering patterns were analyzed using Getis-Ord hot spot analysis, wherein z-scores greater than 2.58 (Bonferroni-corrected *p* < 0.01) signaled high-risk clusters, and z-scores less than −2.58 indicated cold spots (25, 26).

#### Bayesian age-period-cohort model

Furthermore, to forecast future trends in MDR-TB incidence, a BAPC model was implemented in the R statistical environment (version 4.4.3) using the nordpred and BAPC packages. This approach is particularly valuable for understanding the underlying drivers of disease dynamics, as it disentangles the effects of age (reflecting changes in susceptibility across the lifespan), period (capturing population-wide influences such as policies or diagnostics affecting all age groups simultaneously), and birth cohort (representing shared early-life exposures or societal transitions). The BAPC framework is especially suited for producing accurate long-term global projections, as it explicitly accounts for anticipated shifts in the size and age structure of populations—such as aging or demographic transitions across regions—which are critical when forecasting the burden of age-influenced diseases like MDR-TB. This framework decomposed temporal variations into age, period, and cohort effects by employing second-order random walk (RW2) priors for age-specific trends and first-order random walk (RW1) priors for period and cohort effects, with hyperparameters following weakly informative Gamma (1, 0.0005) distributions (27). The model integrated observed age-specific MDR-TB incidence data from 1990 to 2021 with United Nations population projections from 2022 to 2035. Posterior distributions of the parameters were estimated using Markov chain Monte Carlo (MCMC) algorithms, with convergence assessed via Gelman-Rubin diagnostics (R^<1.05) and predictive validity evaluated through leave-one-out cross-validation (LOOCV) (28). The resulting projections were accompanied by 95% credible intervals to quantify uncertainty.

## Results

### Global distribution of MDR-TB burden

**Figure 1:**
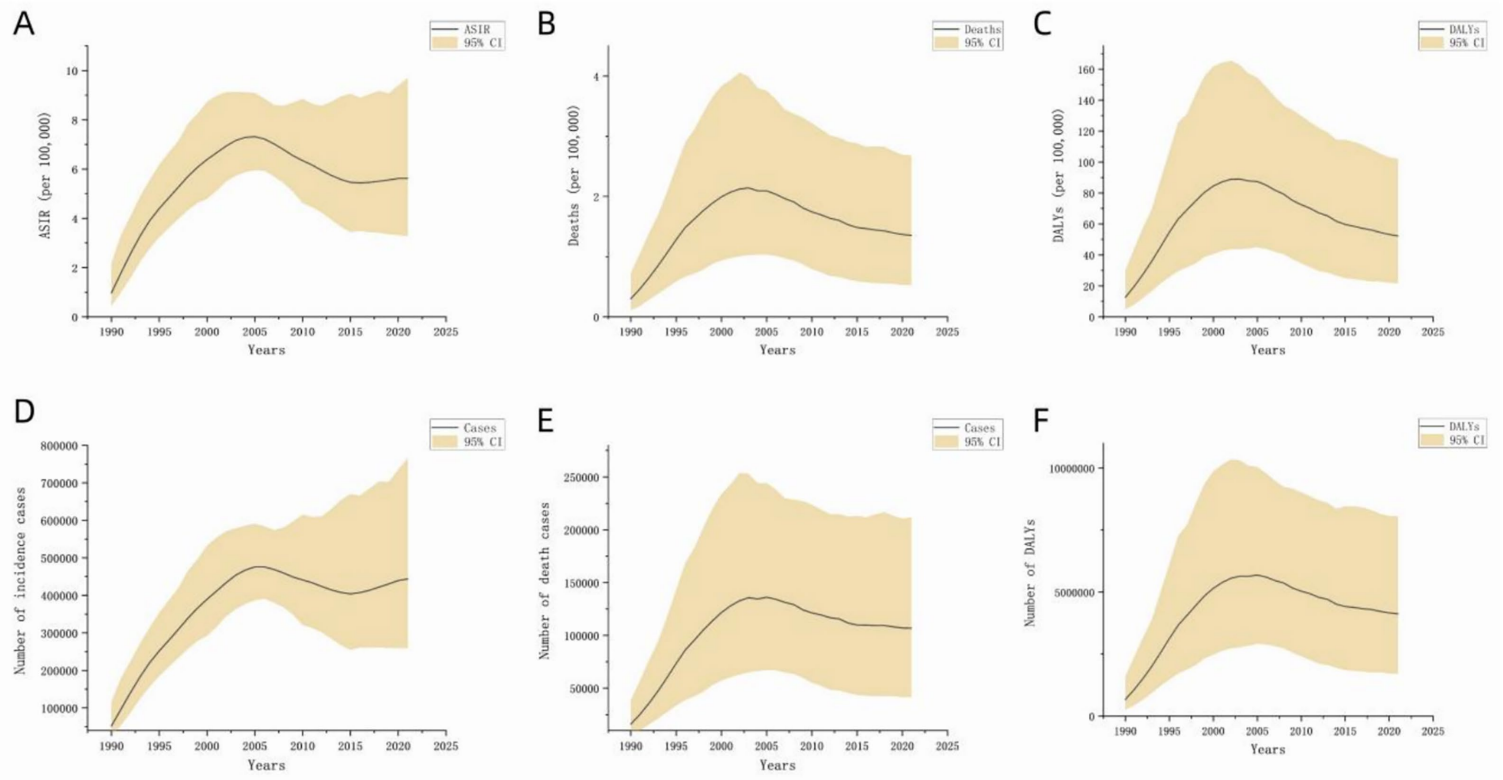
Disease burden of global MDR-TB (1990 to 2021). The black line represents the direction of data distribution. The yellow area represents the upper and lower limits of the 95% confidence interval. **(A)** ASIR, **(B)** ASDR, **(C)** DALYs (per 100,000), **(D)** number of incident cases, **(E)** number of deaths, **(F)** number of DALYs.

**Figure 2:**
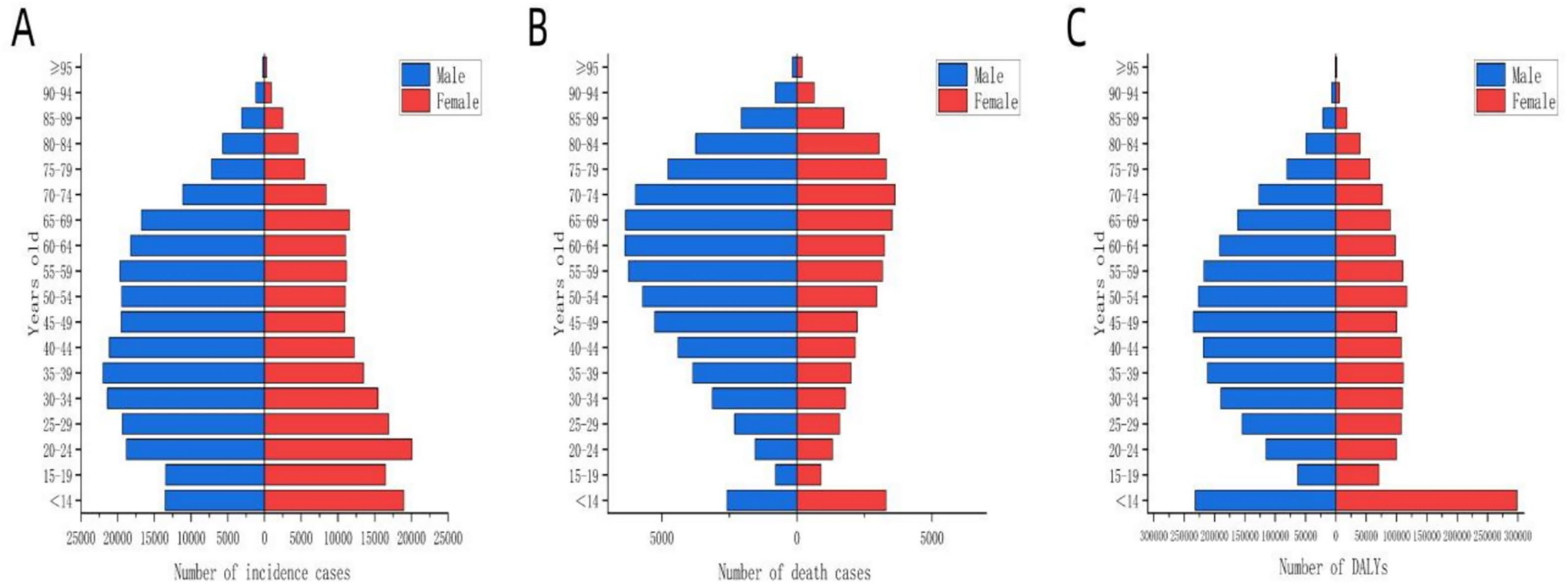
Distribution of disease burden of global MDR-TB by age group for different genders in 2021. The blue bars represent males, and the red bars represent females. **(A)** Number of incident cases, **(B)** number of deaths, **(C)** number of DALYs.

Figure 1 illustrates the global disease burden associated with MDR-TB from 1990 to 2021. Overall, incidence, death, and DALYs initially increased and subsequently decreased over this period. Analysis of the global population pyramid by gender and age (Figure 2) revealed a male-to-female ratio of approximately 1.5:1, with incidence burden primarily concentrated among individuals aged 30 to 60 years, death burden among those aged 60 and above, and DALYs peaking in the 45–60 age group. Notably, the disease burden among children under 14 should not be underestimated.

### Trend of MDR-TB epidemic from 1990 to 2021

**Figure 3:**
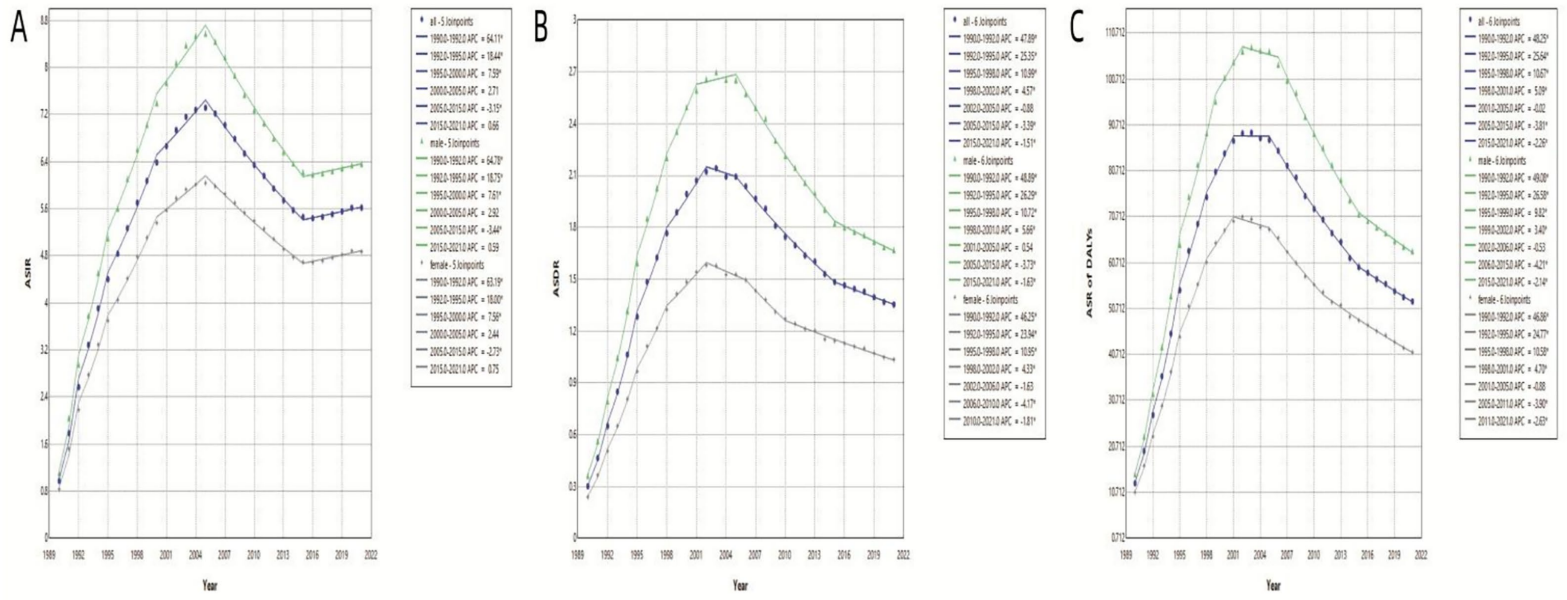
ASIR, ASDR and ASR of DALYs of global MDR-TB based on the joinpoint regression analysis (1990 to 2021). **(A)** ASIR, **(B)** ASDR, **(C)** Age-standardized rate of DALYs.

To elucidate temporal trends, Joinpoint regression analysis identified significant nonlinear trajectories in MDR-TB epidemiology (Table 1). For ASIR, five significant joinpoints (*p*<0.05) were detected over the study period. A rapid increase was observed from 0.97 per 100,000 (95% CI: 0.45–2.20) in 1990 to 6.39 per 100,000 (95% CI: 4.79–8.74) in 2000, followed by a slower increase to 7.32 per 100,000 (95% CI: 5.95–9.10) in 2005. A subsequent decline (2005 to 2015; APC=−3.15, 95% CI: −2.62 to −4.22%) was followed by stabilization at 5.62 per 100,000 in 2021, with a slight upward trend observed from 2016 to 2021. Stratification by gender revealed parallel patterns, with males experiencing a greater increase (+2.39%) and a smaller decrease (−0.71%) compared to females (Figure 3A). ASDR exhibited distinct phases: an initial steep increase from 1990 to 2002 (peak APC=47.89, 95% CI: 45.42–50.44) reaching 2.12 per 100,000 in 2002, followed by stabilization between 2003 and 2005, and a sustained decline thereafter, with 2021 ASDR at 2.12 per 100,000. Notably, male ASDR consistently exceeded that of females, peaking at 2.70 per 100,000 in 2003 (Figure 3B). Similarly, the trend in DALYs mirrored that of ASDR, peaking at 89.05 per 100,000 in 2003 and declining to 52.28 per 100,000 by 2021. Sex-specific trends in DALYs paralleled those observed in death (Figure 3C).

**Table 1:** The epidemic trends of global MDR-TB (1990 to 2021).

Item	Period	APC value	95% CI		*p* value
			Lower	Upper	
ASIR	1990 to 1992	64.11	58.65	69.64	<0.001
	1992 to 1995	18.44	15.43	20.53	<0.001
	1995 to 2000	7.59	5.66	9.44	<0.001
	2000 to 2005	2.71	−1.09	4.30	0.089
	2005 to 2015	−3.15	−4.22	−2.62	0.008
	2015 to 2021	0.66	−0.38	2.22	0.172
ASDR	1990 to 1992	47.89	45.42	50.44	<0.001
	1992 to 1995	25.35	24.13	26.67	<0.001
	1995 to 1998	10.99	9.53	12.01	<0.001
	1998 to 2002	4.57	3.44	5.55	<0.001
	2002 to 2005	−0.88	−2.95	1.37	0.206
	2005 to 2015	−3.39	−4.09	−3.14	<0.001
	2015 to 2021	−1.51	−2.07	−0.67	0.005
DALYs	1990 to 1992	48.25	45.84	50.78	<0.001
	1992 to 1995	25.64	24.45	26.91	<0.001
	1995 to 1998	10.67	9.35	11.70	<0.001
	1998 to 2001	5.09	1.94	6.05	0.004
	2001 to 2005	−0.02	−2.50	0.86	0.777
	2005 to 2015	−3.81	−4.32	−3.53	<0.001
	2015 to 2021	−2.26	−2.79	−1.35	<0.001

APC, annual percent change. CI, Confidence Interval. ASIR, age-standardized incidence rate. ASDR, age-standardized death rate. DALYs, disability-adjusted life years.

### Spatial auto-correlation analysis of MDR-TB incidence

**Figure 4:**
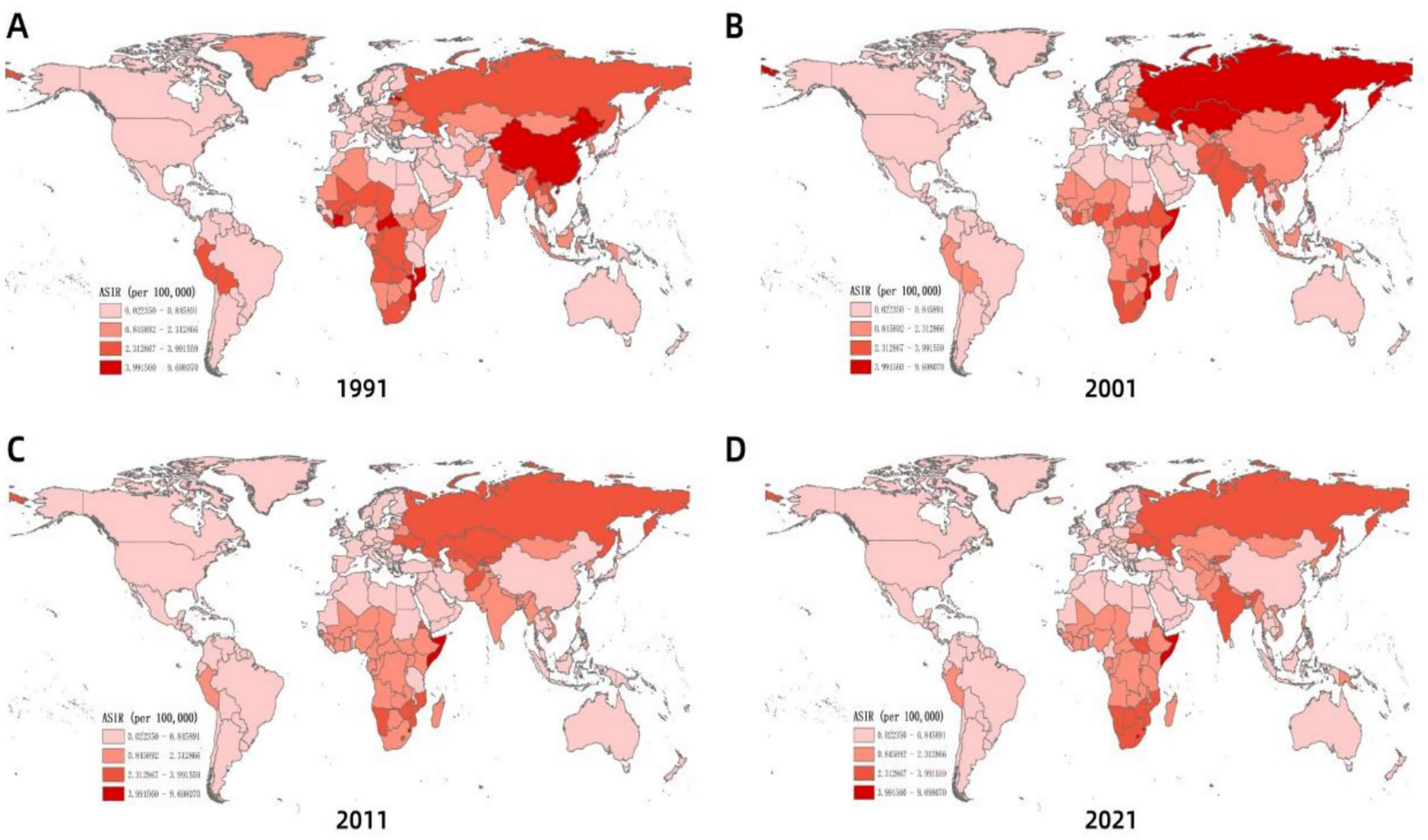
Spatial distribution of ASIR of MDR-TB incidence with years. **(A)** 1991, **(B)** 2001, **(C)** 2011, **(D)** 2021.

**Figure 5:**
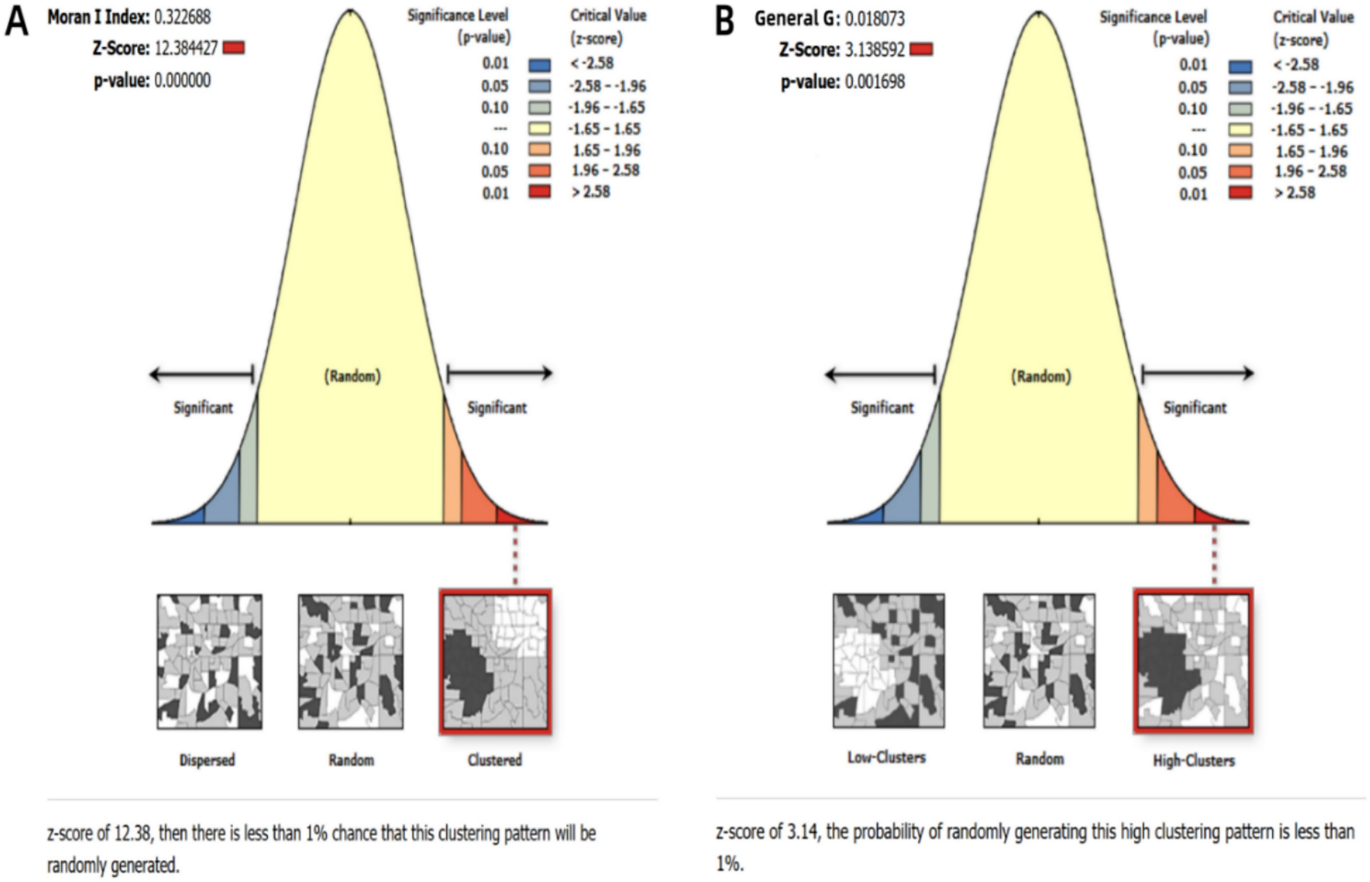
Statistical results of spatial clustering analysis. **(A)** Global spatial autocorrelation analysis; **(B)** Getis-Ord Gi* hotspot analysis.

**Figure 6:**
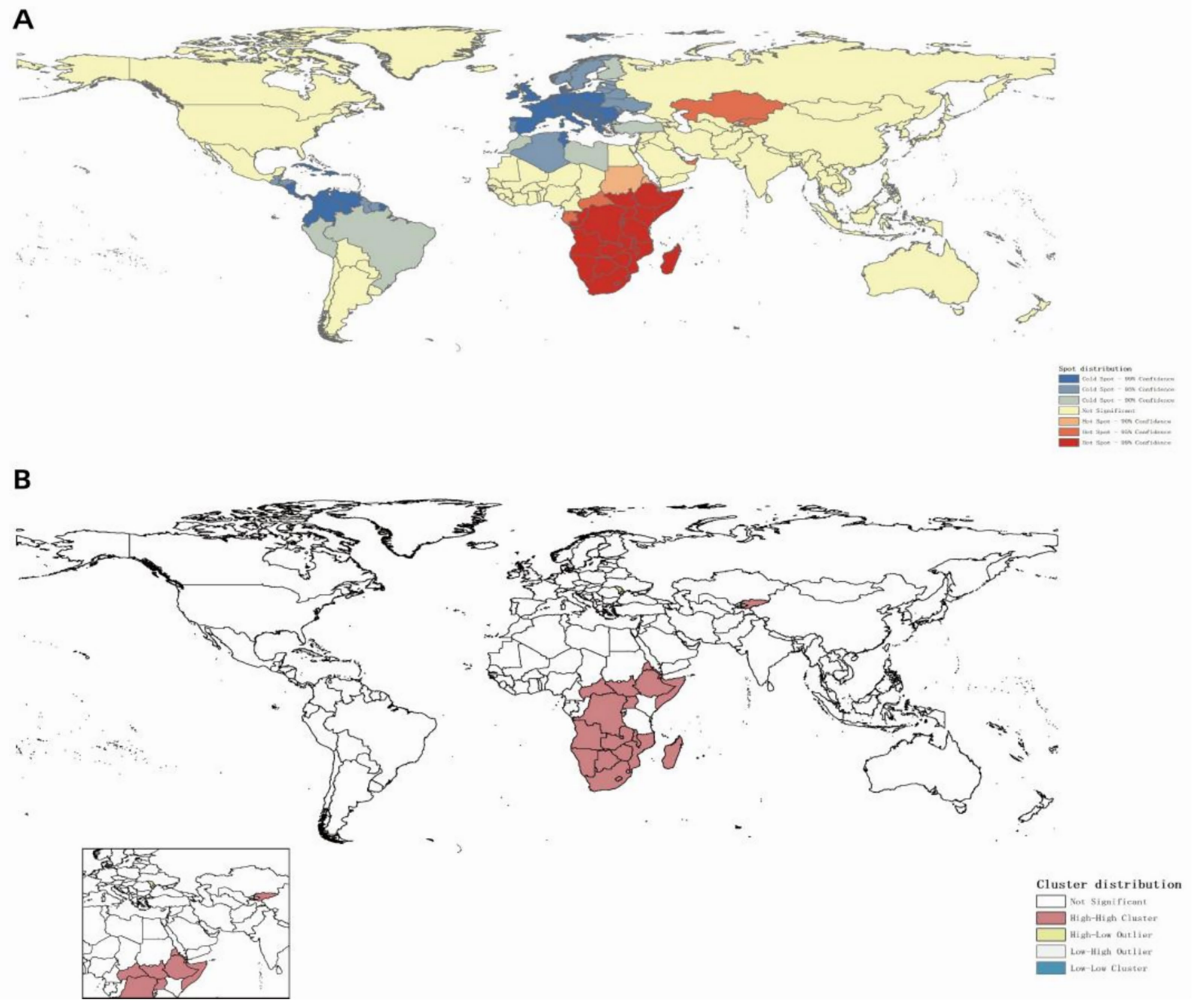
Spatial clustering distribution of MDR-TB incidence (2021). **(A)** Hotspot and coldspot distribution, **(B)** cluster analysis.

Globally, ASIR of MDR-TB exhibits notable geographical heterogeneity (Figure 4). From 1991 to 2021, based on the geographical distributions of MDR-TB ASIR every 10 years, the countries/regions with the highest initial burden ranking of disease include Africa, Russia, India, China, and others. Over time, compared to other countries/regions at the same time, the ranking of MDR-TB outbreaks in countries such as Russia and China has gradually declined, especially in China, with significant changes. On the other hand, in sub-Saharan Africa, the ranking of epidemics in the past 30 years has not changed significantly. High-burden regions are predominantly located in sub-Saharan Africa, India, Central Asia, and Eastern European countries, including Russia. Somalia had the highest ASIR (57.25 per 100,000; 95% CI: 14.12 to 169.56), with 11 countries or territories recording rates exceeding 20 per 100,000. Significant spatial correlation was confirmed by Moran’s I index (z=12.38, *p*<0.001), indicating regional clustering (Figure 5A). Hot spot analysis revealed marked spatial aggregation, with primary hotspots situated in Central and Southern Africa and cold spots identified in Europe, North/Central America, the Caribbean, and northern South America (Figure 6A). Cluster mapping detected two high-high clusters: one forming a contiguous zone across sub-Saharan Africa and another isolated cluster in Kyrgyzstan (z=3.14, *p*=0.002; Figure 5B). No statistically significant low clusters were observed (Figure 6B).

### Projection of MDR-TB incidence from 2022 to 2035

**Figure 7:**
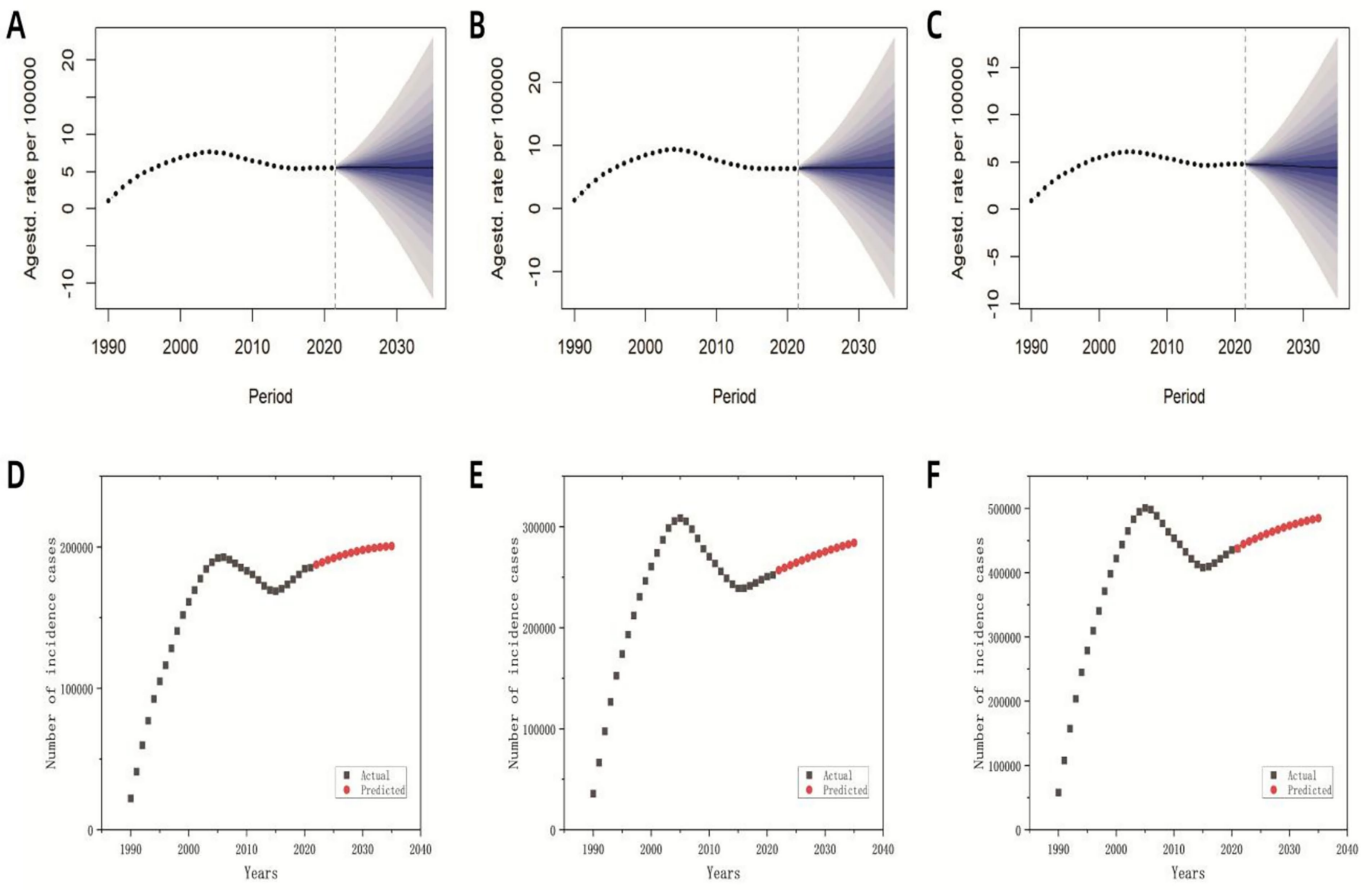
Predicted global incidence rates and case numbers for MDR-TB based on the BAPC model (2022–2035). **(A)** The predicted overall incidence rate; **(B)** the predicted incidence rate for males; **(C)** the predicted incidence rate for females; **(D)** the predicted overall number of incident cases; **(E)** the predicted number of incident cases among males; **(F)** the predicted number of incident cases among females.

Based on historical MDR-TB incidence data spanning 1990 to 2021 and age-stratified population projections from the WPP 2024, we employed a BAPC model to forecast global MDR-TB incidence through 2035. Although Joinpoint regression previously identified a decline post-2005 followed by stabilization, our projections suggest persistent challenges. Incidence is expected to increase slightly from 2022 to 2025 (to 5.55 per 100,000; 95% CI: 2.41 to 8.63), with a turning point in 2026 that initiates gradual declines. By 2035, the incidence is projected to remain elevated at 5.46 per 100,000 (95% prediction interval: −12.01 to 22.88), representing a reduction of only 1.62% relative to 2025 levels (Figure 7A). Although male incidence trajectories mirror overall trends, female rates are projected to decrease by 7.27% compared to 2025 (Figures 7B,C). Despite this modest decline, projected global population growth to 8.89 billion by 2035 is expected to drive an increasing absolute case count, with annual MDR-TB cases are projected to reach over 480,000 by 2035 (Figure 7D). Among these cases, approximately 280,000 are expected to occur in males and 200,000 in females (Figures 7E,F).

## Discussion

This study provides for the first time, a comprehensive spatiotemporal epidemiological picture of global MDR-TB spanning 46 years (1990–2035), filling the knowledge gap regarding its long-term evolution patterns, population and geographic heterogeneity, and refined long-term predictions. Our analysis confirmed and quantified the non-linear changes in MDR-TB burden over time, significant demographic differences, and persistent geographic clustering. Crucially, the BAPC model predicts that the global burden of MDR-TB will remain high until 2035, especially with population growth and aging, the absolute number of cases is projected to increase. This finding posed a serious challenge to achieving the End TB target.

The widespread adoption of first-line anti-TB drugs (isoniazid and rifampicin) since the 1980s, particularly after rifampicin’s inclusion in the short-course regimen that reduced treatment duration from 18–24 months to 6 months, was initially transformative (29, 30). However, these gains were eroded by escalating drug resistance, driven by suboptimal adherence to prolonged regimens and inadequate drug exposure in settings with weak health systems (31, 32). The steady increase in ASIR between 1990 and 2005 reflects both the expansion of drug-resistant TB transmission and historical diagnostic limitations. Late-twentieth-century improvements in diagnostic methodologies—including the advent of phenotypic techniques (e.g., solid culture drug susceptibility testing) and molecular diagnostics (e.g., Xpert MTB/RIF) (33, 34)—significantly enhanced case detection, thereby revealing previously undiagnosed reservoirs. The subsequent decline in ASIR post-2005 (annual percentage change = −3.15%) is likely associated with the implementation of enhanced TB control strategies, such as the directly observed treatment strategy (DOTS), and improved access to second-line therapies (35, 36). However, the recent plateau and slight resurgence in incidence since 2016 underscore persistent systemic gaps; treatment success rates remain at approximately 50%, thereby perpetuating community transmission of resistant strains. The impact of the COVID-19 pandemic on the global healthcare industry may have halted the downward trend of MDR-TB incidence after 2019. A review covering 17 low- and middle-income countries (LMICs) shows that the number of MDR-TB cases rebounded after the pandemic, especially in areas with weak healthcare infrastructure such as Indonesia and India (37). Concurrent declines in ASDR and DALYs from 2015 to 2021, as reported in global TB studies (38), suggest partial effectiveness of intensified MDR-TB management efforts. Nonetheless, persistent incidence trends necessitate the urgent adoption of novel transmission control strategies, optimized treatment regimens (e.g., incorporating bedaquiline and pretomanid), and the scaling up of quality assurance programs to effectively curb further transmission.

Our research indicated that the global burden of MDR-TB exhibits marked gender and age stratification. Notably, there were pronounced gender disparities, with males exhibiting substantially higher ASIR, ASDR and DALYs than females. This sex-based gradient aligned with established tuberculosis epidemiology and likely reflects male-predominant risk factors such as smoking, alcohol consumption, occupational exposures (e.g., mining or migrant labor), delayed healthcare-seeking behaviors, and sub-optimal living conditions (8). Collectively, these factors heightened resistance risks (39, 40) and underscore the importance of implementing gender-sensitive interventions—particularly community-based active screening targeting male populations—to disrupt transmission asymmetries (41). The incidence number burden was concentrated among middle-aged individuals, primarily those between 30 and 60 years of age (42), a trend that might be related to frequent social interactions, occupational exposures, and histories of nonstandard treatment among young and middle-aged populations (8, 43). In contrast, the death burden was predominantly observed in the older adults, specifically among individuals aged 60 years and above. For instance, a study in South Korea reported that patients over 75 years of age had a death risk 68 times higher than those under 24, with individuals over 65 accounting for 65% of total deaths (44). Additionally, DALYs peaked in the 45–60 age group, reflecting significant labor loss and socioeconomic impact (9). The burden among children under 14 years of age should not be underestimated. It was estimated that annually, between 25,000 and 32,000 children contract MDR-TB—accounting for approximately 3% of children’s tuberculosis cases—yet only 3–4% of these children receive standard treatment, resulting in a 21% death (45). Insufficient diagnostic capacity was a core issue, compounded by limited treatment options: while a 6–9-month short-course regimen was effective in adults, appropriate dosage forms and safety data for children were lacking (46). Strengthening the management of family contacts, developing child-friendly formulations, and incorporating children into pediatric clinical trials are essential strategies for reducing the disease burden in this vulnerable population (47).

Geospatial clustering analyses identified hyperendemic MDR-TB zones in sub-Saharan Africa, Central Asia, India, and Russia. In these regions, syndemic interactions with HIV/AIDS—reflected in co-infection rates of 20% in Africa—fragile health systems, poverty, and armed conflicts perpetuate transmission (48, 49). In South Africa, one-third of diagnosed TB cases discontinue treatment, thereby fueling the propagation of MDR-TB (50, 51). Moreover, in conflict zones such as Somalia, the collapse of healthcare infrastructure has created persistent hotspots through recurrent treatment interruptions (52). In contrast, European cold spots were associated with robust socioeconomic development and universal healthcare access. Through free treatment, transportation subsidies, and occupational protection policies in Europe and other regions, TB patients do not need to interrupt treatment due to economic pressure, effectively blocking the transmission chain of drug-resistant bacteria in the community (53). To mitigate the occurrence of MDR-TB, it was imperative to implement social protection and poverty reduction strategies in low-income regions with lagging socioeconomic development (54), alongside increased allocation of medical resources to local populations. Phlegm culture, genetic testing (such as Xpert MTB/RIF), and second-line drug costs should be covered, encompassing the entire process from diagnosis to treatment. We strongly urged the international community to make targeted, strengthened, and sustainable investments in the region, such as enhancing diagnostic capabilities, promoting short-term programs, addressing HIV comorbidities, and providing social support.

The BAPC model projected a marginal 1.62% decline in global MDR-TB incidence by 2035, despite an anticipated increase to 480,000 cases annually. These findings, which align with Sharma’s earlier projections (7), suggested that high-burden nations such as India and Russia will continue to experience rising incidence until 2040, driven by demographic expansion and the accumulation of drug-resistant reservoirs. The core engine of future global population growth is sub Saharan Africa (contributing over 50% of the new population), followed by South Asian countries such as India and Pakistan (55). This trend is driven by ultra-high fertility rates, a young population structure, and a lagging transition in fertility. The proportion of older adults aged 65 and above will continue to rise, expected to reach 16% by 2050 (56). This underscores the need to prioritize absolute disease burden metrics in MDR-TB surveillance, especially as the gap with the WHO’s “End TB” targets widens due to multi-factorial drivers including demographic growth, imbalanced healthcare resource distribution, and cross-border transmission of resistant strains (9, 57). Although a six-month short treatment regimen (BPaL/M) has demonstrated improved outcomes, its prohibitive cost has limited accessibility for MDR-TB patients in low-income settings (58). To address these challenges, it was recommended that the WHO and national governments develop targeted strategies for low-resource regions, consider implementing initiatives such as “government subsidies + healthcare” to alleviate catastrophic treatment costs and ensure comprehensive patient care and support (59). A study shows that the local government of the Republic of Congo has taken measures to strengthen molecular monitoring and control to manage drug-resistant TB (60). A study in Brazil shows that measures such as reducing population density and controlling HIV can reduce the incidence rate of drug-resistant TB (61).

This study has limitations. First, we relied on GBD estimated data, which may under- or overestimate the true MDR-TB burden due to variable data quality across countries. Second, the BAPC model assumes future trends mirror historical patterns, overlooking disruptive events like pandemics, conflicts, or medical breakthroughs. Third, lacking global socioeconomic data restricts our analysis of factors driving disease burden disparities. Future work should integrate real-time surveillance with dynamic models and assess socioeconomic determinants of MDR-TB.

## Conclusion

This first comprehensive spatiotemporal analysis spanning 46 years reveals that the MDR-TB epidemic presents a far more formidable challenge to the End TB goals than previously appreciated. It is characterized by persistent geographical hotspots (notably in sub-Saharan Africa), a disproportionate burden among males, the older adults, and children, and a projected rise in absolute cases to 480,000 by 2035—despite a marginal decline in ASIR—driven by sustained population growth and aging, presents a far more formidable and enduring challenge to the End TB goals than previously appreciated from fragmented or shorter-term studies. Effectively mitigating this trend demands a paradigm shift: (1) Hyper-targeted resource allocation informed by spatial hotspot mapping to sub-Saharan Africa and other high-burden clusters; (2) Development and implementation of demographically-tailored interventions addressing the specific barriers faced by men (e.g., strengthen management and intervene in behavior), the older adults (e.g., active case finding in high-risk settings, integrated care), and children (e.g., improved diagnostics, child-friendly formulations, contact investigation); (3) Sustained investment in novel tools and strategies (shorter regimens, new drugs, vaccines); (4) Strengthen government commitments, provide patients with more economic support, implement “government subsidies + healthcare” initiatives to reduce the burden of diagnosis and treatment; (5) Carry out international regional cooperation to prevent and control MDR-TB through assistance programs. Resource allocation and effective healthcare interventions in the face of this persistent threat would galvanize more urgent, focused, and equitable global action.

## Data Availability

The raw data supporting the conclusions of this article will be made available by the authors, without undue reservation.

## Footnotes

^1^ http://ghdx.healthdata.org/gbd-results-tool

^2^ https://population.un.org/wpp

^3^ https://seer.cancer.gov/stdpopulations/world.who.html

^4^ https://www.naturalearthdata.com
